# Naringin alleviates gefitinib-induced hepatotoxicity through anti-oxidation, inhibition of apoptosis, and autophagy

**DOI:** 10.22038/ijbms.2024.76852.16623

**Published:** 2024

**Authors:** Dan Liu, Changlin Zhen, Xiuzhen He, Wansong Chen, Juan Pan, Mengying Yin, Mengru Zhong, Hongyan Zhang, Xiaohuan Huang, Yonghui Zhang

**Affiliations:** 1 Chongqing Key Laboratory of Development and Utilization of Genuine Medicinal Materials in Three Gorges Reservoir Area, Department of Basic Medicine, Chongqing Three Gorges Medical College, Chongqing 404120, China

**Keywords:** Apoptosis, Autophagy, Gefitinib, Hepatotoxicity, Naringin

## Abstract

**Objective(s):**

Gefitinib (GEF) is a targeted medicine used to treat locally advanced or metastatic non-small cell lung cancer (NSCLC). However, GEF’s hepatotoxicity limits its clinical use. This study aims to investigate the protective effect of naringin (NG) against GEF-induced hepatotoxicity.

**Materials and Methods:**

Fifty female ICR mice were randomly divided into 5 groups: Control, GEF (200 mg/kg), NG (50 mg/kg) + GEF (200 mg/kg), NG (100 mg/kg) +GEF (200 mg/kg), NG (200 mg/kg) +GEF (200 mg/kg). After 4 weeks of continuous administration, the mice were euthanized. The blood and liver tissue samples were collected.

**Results:**

The results indicated that the GEF group showed increased liver index, liver enzyme activities, and decreased glutathione (GSH), superoxide dismutase (SOD), and catalase (CAT) activities. Some hepatocytes showed hydropic degeneration and focal necrosis. Cell apoptosis, Cleaved-caspase3, and Poly (ADP-ribose) polymerase 1 (PARP1) increased. Transmission electron microscopy revealed the presence of numerous autophagic lysosomes or autophagosomes around the cell nucleus. Compared to the GEF group, NG can reverse these changes.

**Conclusion:**

In summary, NG alleviates GEF-induced hepatotoxicity by anti-oxidation, inhibiting cell apoptosis, and autophagy. Therefore, this study suggests the use of NG to mitigate GEF’s toxicity to the liver.

## Introduction

Selective epidermal growth factor receptor-tyrosine kinase inhibitors (EGFR-TKIs) are effective in treating NSCLC with EGFR mutations (1). Clinical observations have shown that long-term oral administration of EGFR-TKIs can lead to hepatotoxicity. Patients with pre-existing hepatitis virus infections or liver damage have an increased risk of hepatotoxicity when taking EGFR-TKIs. Gefitinib (GEF) has a more significant risk of hepatotoxicity compared to icolitinib/erlotinib (2). Among 152 patients receiving GEF treatment, 52 (34.2%) experienced GEF-induced hepatotoxicity (3). One study found that 24% to 30% of patients receiving GEF treatment needed to discontinue therapy due to hepatotoxicity (4, 5). In one case, a patient with EGFR mutation-positive lung adenocarcinoma taking GEF 250 mg daily for 16 weeks experienced a significant increase in serum liver enzymes (Alanine aminotransferase and aspartate aminotransferase). However, after switching to the second-generation EGFR-TKI, afatinib, for at least 44 weeks, the patient did not suffer from hepatotoxicity or disease progression (6). Clinical strategies for addressing hepatotoxicity include switching from first-generation EGFR-TKIs to second-generation EGFR-TKIs, which has improved hepatotoxicity in some patients. However, clinical trials have shown that second-generation EGFR-TKI afatinib can also cause increased alanine aminotransferase (ALT) and aspartate aminotransferase (AST) levels, leading to liver damage (7, 8). Discontinuation of treatment can accelerate tumor growth. Therefore, there is an urgent need to find natural, non-toxic agents that can mitigate GEF-induced hepatotoxicity.

NG is a natural flavonoid compound primarily found in the peel of citrus fruits such as pomelos, oranges, lime, grapefruit, and varieties. Research has confirmed that NG possesses various pharmacological properties, including anti-inflammatory, anti-oxidant, and hepatoprotective effects (9). NG has been shown to protect against diclofenac-induced hepatotoxicity by reducing ALT, AST, lactate dehydrogenase (LDH), alkaline phosphatase (ALP), total bilirubin, and tumor necrosis factor-α (TNF-α) levels (10). Studies have found that NG protects against cyclophosphamide (CYCP)-induced hepatotoxicity and nephrotoxicity by regulating oxidative stress, inflammation, cell apoptosis, autophagy, and DNA damage (11). Further research has revealed that NG pretreatment significantly reduces CYCP-induced elevation of ALT, AST, and LDH activities in serum and liver tissue, as well as decreases the content of malondialdehyde (MDA), hydrogen peroxide (H_2_O_2_), and nitric oxide (NO). NG also reverses the reduction in CYCP-induced hepatic glutathione (GSH) levels, superoxide dismutase (SOD) activity, and the down-regulation of catalase (CAT), glutathione peroxidase (GPx), and glutathione reductase (GR) activities. It also attenuates the up-regulation of CYCP-induced chemokine ligand 2 (CCL_2_), interferon-α1 (IFN-α1), interleukin-1β, interleukin-1 receptor, and transforming growth factor β1 (TGF-β1) expression. This suggests that NG prevents CYCP-induced rat hepatotoxicity by reducing oxidative stress, fibrosis, and inflammation (12). Compared to the 5-fluorouracil (5-FU)-induced hepatotoxicity model group, the NG-administered group showed significant restoration of SOD and GSH levels and a decrease in liver enzymes and MDA levels. NG significantly alleviated 5-FU-induced hepatotoxicity by restoring cellular anti-oxidant capacity. NG exhibits potent protective effects against drug-induced liver injury, primarily achieved by up-regulating nuclear factor erythroid-2-related factor 2 (Nrf2) and down-regulating nuclear factor kappa B (NF-κB) to exert its anti-oxidant, anti-inflammatory, and anti-apoptotic effects (13). However, the impact of NG on GEF-induced liver damage is currently unclear.

Therefore, our research group will establish a mouse model of GEF-induced hepatotoxicity and confirm the protective effect of NG on GEF-induced hepatotoxicity through liver index measurements, blood biochemical indicators, and anti-oxidant index and hepatic histopathological examination using hematoxylin and eosin (HE) staining. We will further use the terminal deoxynucleotidyl transferase dUTP nick-end labeling (TUNEL) method to detect hepatocyte apoptosis and transmission electron microscopy to examine autophagosomes and autolysosomes. In addition, we will use immunohistochemistry to detect apoptosis-related proteins cleaved-caspase3 and PARP1. This study aims to demonstrate that NG protects against GEF-induced hepatotoxicity by anti-oxidation, inhibiting cell apoptosis, and autophagy.

## Materials and Methods

***Animals***

GEF was obtained from Shanghai yuanye Bio-Technology Co., Ltd (Shanghai, China). NG was produced by Sigma-Aldrich Company (St. Louis, MO, USA). ALT, AST, LDH, ALP, GSH, SOD, and CAT activity assay kits were purchased from Nanjing Jiancheng Bioengineering Institute (Nanjing, China). Cleaved-caspase3 and PARP1 were purchased from Servicebio (Wuhan, China). All other chemicals used were of analytical grade and were obtained from local chemical companies.

***Experimental animals***

SPF healthy female ICR mice, 5–6 weeks old, weighing 18–22 g, were purchased from Hunan SJA Laboratory Animal Co., Ltd (Changsha, China) (Production license No.: SCXK (Xiang) 2019-0004). The animal experiment was carried out in the Animal Experiment Center of Chongqing Three Gorges Medical College, reared in accordance with the regulations of the National Laboratory Animal Breeding and Management, and approved by the Medical Ethics Committee of Chongqing Three Gorges Medical College (approval number: SXYZ-A-2308-0002). The animals were given one week to acclimate to the laboratory environment (12-hour light/dark cycle, 26–28 °C, relative humidity 40%–60%) before the start of the experiment.

***Animal groups***

**Figure 1:**
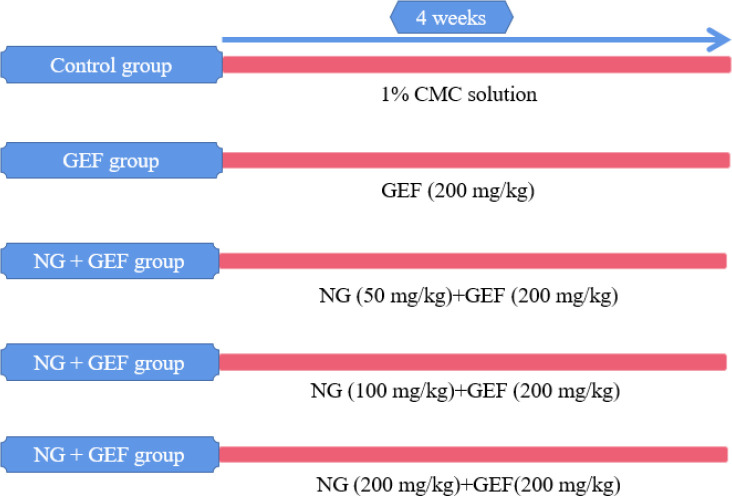
Experimental design and animal grouping

NG: Naringin; GEF: Gefitinib

Fifty female ICR mice were divided into 5 groups (Figure 1).

Group 1 (Control) received daily oral gavage of an equivalent volume of 1% carboxymethyl cellulose sodium (CMC; vehicle for dissolving GEF and NG).

Group 2 (GEF) received oral GEF (dissolved in 1% CMC) at a dose of 200 mg/kg/day.

Group 3 (NG50 + GEF) received GEF as in Group 2 and oral NG (dissolved in 1% CMC) at a dose of 50 mg/kg/day.

Group 4 (NG100 + GEF) received GEF as in Group 2 and oral NG (dissolved in 1% CMC) at a dose of 100 mg/kg/day.

Group 5 (NG200 + GEF) received GEF as in Group 2 and oral NG (dissolved in 1% CMC) at a dose of 200 mg/kg/day.

Each group received continuous gavage for 4 weeks.

***Serum preparation***

At the end of the experiment, blood was collected from the mice by eyeball enucleation. Blood samples were allowed to stand at room temperature for 1-2 hr, followed by centrifugation at 3000 rpm for 10 min to obtain serum. Serum from each blood sample was divided into three small vials and stored at -80 °C for biochemical analysis. After euthanasia, liver tissue from the right lobe was fixed in 4% paraformaldehyde for observation of liver morphology, detection of cell apoptosis by TUNEL assay, and immunohistochemical detection (5). Liver tissue pieces (2 mm x 2 mm) were fixed with an electron microscope fixative for observation by transmission electron microscopy. 

***Calculation of mouse liver index***

Throughout the experiment, daily observations were made on the mental state, activity, food intake, and water consumption of the experimental mice. The body weight of each mouse before euthanasia was recorded and the liver was removed and reweighed to calculate the liver index.

Calculation formula: Liver Index = Liver Mass / Mouse Body Mass × 100%.

***Measurement of liver function parameters in serum***

Serum levels of ALT, AST, LDH, and ALP activities were measured according to the instructions of the respective test kits. 

***Measurement of the anti-oxidant indexes in the liver tissue***

Liver tissue levels of GSH, SOD, and CAT activities were measured according to the instructions of the respective test kits. 

***Histopathological examination of liver tissues***

Right lobe liver tissues were fixed in 4% paraformaldehyde for 24 hr, embedded in paraffin, sectioned into 4 μm slices, and stained with HE (14). Pathological changes in liver tissue were observed under an optical microscope, and images were captured.

***Detection of hepatocyte apoptosis by TUNEL assay***

Paraffin sections of mouse liver tissue were deparaffinized in xylene for 10 min, 1% protease K drops were added to the tissue and incubated in a 37 °C incubator for 22 min, covered with 0.1% triton, and incubated at room temperature for 20 min, covered with buffer and incubated at room temperature for 10 min. After washing with buffer, sections were incubated with the TUNEL reaction mixture (TDT enzyme: dUTP: buffer in a 1: 5: 50 ratio) in a 37 °C incubator for 2 hr. DAPI staining solution was added and incubated at room temperature for 10 min. Liver tissue sections were sealed and observed under a fluorescence microscope and images were collected (15).

***Cleaved-caspase3 and PARP1 protein expression by immunohistochemical staining***

Paraffin sections were deparaffinized and heat-treated in citrate buffer in a microwave for 10 min, followed by washing three times in PBS. Endogenous peroxidase was blocked by incubation in 3% hydrogen peroxide for 25 min away from light at room temperature. Sections were blocked with 3% BSA for 30 min at room temperature and then incubated with antibodies cleaved-caspase3 (1: 500) and PARP1 (1:1000) overnight at 4 °C. Tagged with HRP, goat anti-mouse IgG, secondary antibody, was added and incubated at 37 °C for 10 min. Adding DAB color-developing solution was followed by counterstaining with hematoxylin for 5 min, and the sections were dehydrated to transparency, cleared in xylene for 6 min, and sealed. Images were collected under an optical microscope. 

***Observation of autophagosomes and autolysosomes by transmission electron microscopy***

Liver tissue pieces (2 mm x 2 mm) were fixed with an electron microscope fixative for observation by transmission electron microscope. After fixation with 2.5% glutaraldehyde and osmic acid, tissue samples were dehydrated with conventional acetone, embedded in resin, and ultra-thin sections were cut. Sections were double-stained with 3% uranyl acetate and lead citrate. Prepared samples were observed for the ultrastructure of autophagosomes and autolysosomes in liver tissue using transmission electron microscopy.

***Statistical analysis***

Data were analyzed using one-way analysis of variance (ANOVA) in GraphPad Prism 5.0 (GraphPad, USA). Data are presented as means ± standard deviation (SD) for ten mice in each group. Differences were considered significant at *P*<0.05.

**Figure 2:**
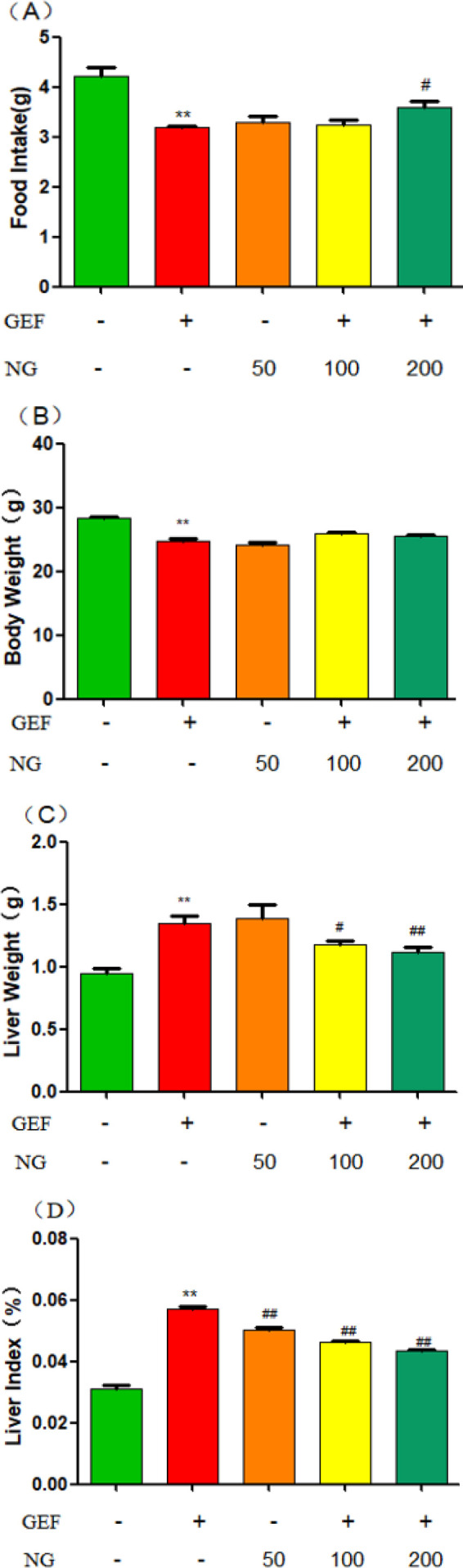
Effect of NG on food intake, body weight, liver weight, and liver index in the ICR mice of GEF-induced liver injury

(A) Food Intake, (B) Body Weight, (C) Liver Weight, (D) Liver index

Each bar represents the mean ± SD of 10 animals in each group; Compared with control group, **P<0.01, Compared with GEF group, #*P*<0.05, ##*P*<0.01

NG: Naringin; GEF: Gefitinib

**Figure 3:**
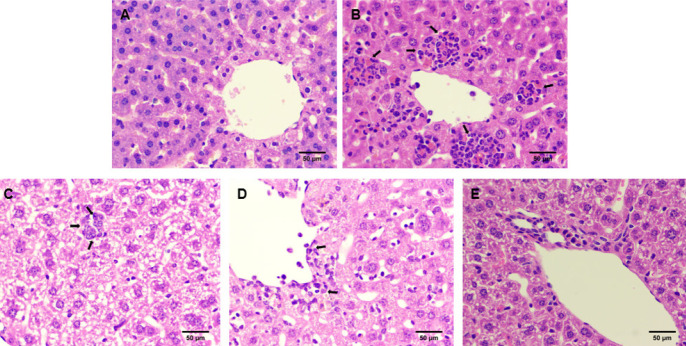
The effect of NG on liver tissue of mice with GEF-induced liver injury

Liver histopathology showed micrographs of liver sections from the (A) Control, (B) GEF, (C) NG50 + GEF, (D) NG100 + GEF, and (E) NG200 + GEF groups. Neutrophil infiltration and focal necrosis are indicated by black arrowheads (H&E; ×400).

GEF: Gefitinib; NG: Naringin

**Figure 4:**
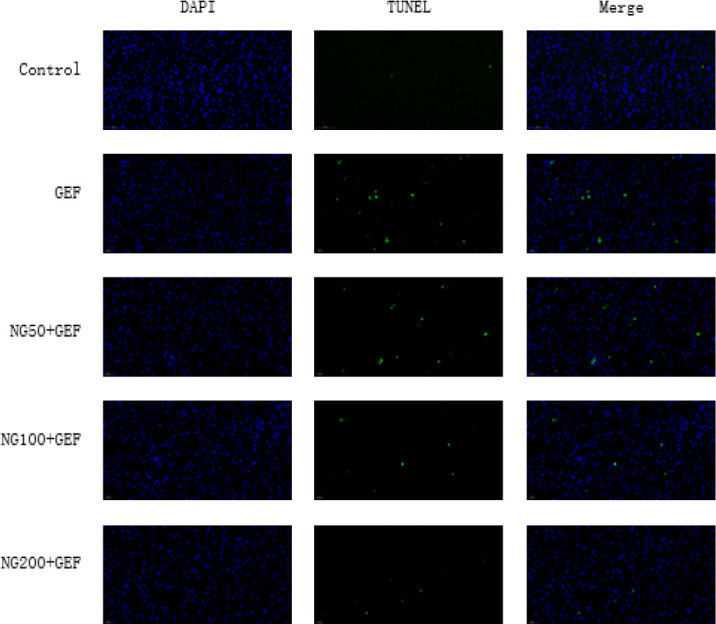
Effect of NG on apoptosis in liver tissue sections (TUNEL staining) of ICR mice with GEF-induced liver injury

GEF: Gefitinib; NG: Naringin

**Figure 5:**
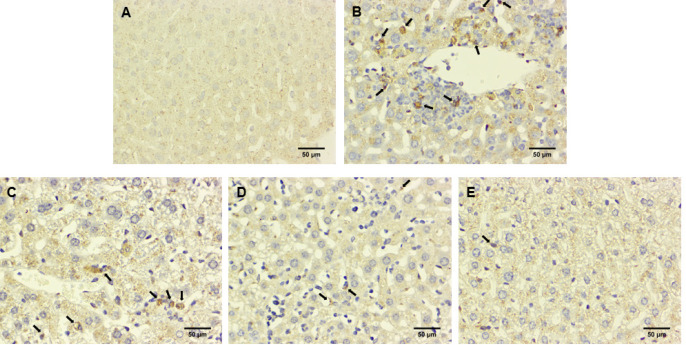
Effect of NG on the expression of cleaved-caspase 3 in liver tissue of ICR mice with GEF-induced liver injury

(A) Control group, (B) GEF group showing severe positive response, (C) NG50 + GEF group showing moderate positive response, (D) NG100 + GEF, and (E) NG200 + GEF groups showing mild positive response (×400).

GEF: Gefitinib; NG: Naringin

**Figure 6:**
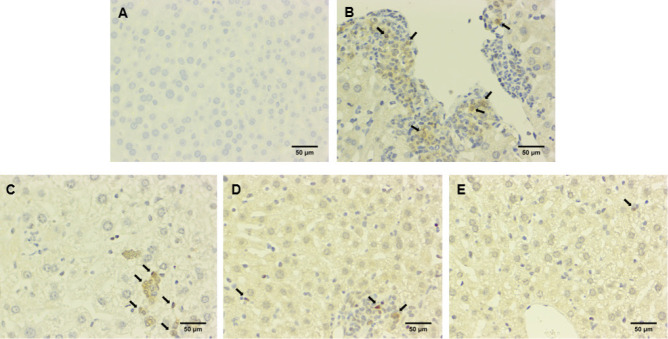
Effect of NG on the expression of PARP1 in liver tissue of ICR mice with GEF-induced liver injury

(A) Control group, (B) GEF group showing severe positive response, (C) NG50 + GEF, (D) NG100 + GEF groups showing moderate positive response, and (E) NG 200 + GEF group showing mild positive response (×400).

PARP1: Polymerase 1; GEF: Gefitinib; NG: Naringin

**Figure 7:**
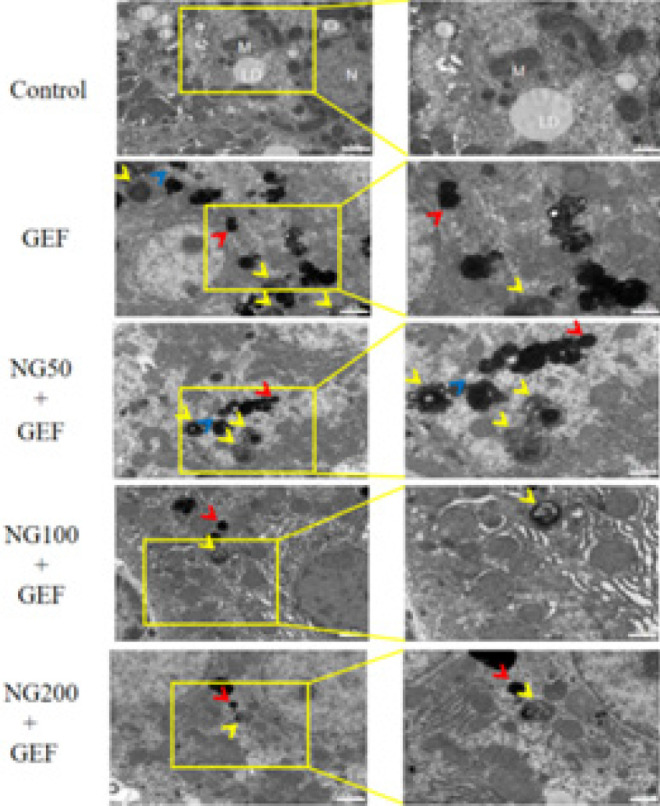
Transmission electron microscopy results and statistical analysis

Note: N represents the cell nucleus; M represents mitochondria; LD represents lipid droplets; yellow arrows indicate autolysosomes; red arrows indicate lysosomes; blue arrows indicate autophagosomes.

**Table 1:** Effect of NG on hepatic function-related biochemical parameters in the serum of ICR mice with GEF-induced liver injury (mean ± SD), n = 10

Group	ALT (U/L)	AST（U/L）	LDH (U/L)	ALP (U/L)
Control	27.77±3.01	67.67±11.56	5.39±0.435	129.5±21.01
GEF	175.60±12.72^**^	341.0±25.73^**^	16.26±0.871^**^	476.0±24.35^**^
NG 50+GEF	122.90±14.53^#^	226.5±21.46^##^	11.44±1.032^#^	372.9±30.06^##^
NG 100+GEF	83.07±8.02^##^	135.7±17.07^##^	7.72±0.447^##^	301.6±29.98^##^
NG 200+GEF	51.85±3.36^##^	61.71±14.07^##^	6.43±0.72^##^	241.6±29.06^##^

Compared with control group,*P<0.05,**P<0.01, Compared with GEF group, #*P*<0.05, ##*P*<0.01

GEF: Gefitinib; NG: Naringin; ALT: Alanine aminotransferase; AST: Aspartate aminotransferase; LDH: Lactate dehydrogenase; ALP: Alkaline phosphatase

**Table 2:** Effect of NG on anti-oxidant parameters of liver tissue of ICR mice with GEF-induced liver injury (mean ± SD), n = 8

Group	GSH（μmol/gprot）	SOD（U/L）	CAT (U/L)
Control	147.30±21.54	18.01±1.35	20.74±2.92
GEF	107.24±17.43^*^	14.40±1.53^**^	16.93±4.59
NG100+GEF	128.27±12.80^#^	23.28±2.72^##^	23.94±4.60^#^

Compared with control group, *P<0.05, **P<0.01; Compared with GEF group, #*P*<0.05, ##*P*<0.01

GEF: Gefitinib; NG: Naringin; SOD: Superoxide dismutase; GSH: Glutathione; CAT: Catalase

**Table 3:** Histopathological evaluation of liver tissue of ICR mice with GEF-induced liver injury

Parameters	Control	GEF	NG50 + GEF	NG100 + GEF	NG200 + GEF
Hydropic degeneration in hepatocytes	−	+	+++	++	+
Focal necrosis	−	+++	+	++	+
Neutrophils infiltration	−	+++	+	++	+

(−) No variation, (+) Small variation, (++) Medium variation, (+++) Big variation

GEF: Gefitinib; NG: Naringin

**Table 4:** The evaluation of cleaved-caspase3, PARP1 and LC3B in liver tissue of ICR mice with GEF-induced liver injury

Parameters	Control	GEF	NG50 + GEF	NG100 + GEF	NG200 + GEF
Cleaved-caspase3	−	+++	++	+	+
PARP1	−	+++	++	+	+

(−) No variation, (+) Small variation, (++) Medium variation, (+++) Big variation. GEF: Gefitinib; NG: Naringin

**Table 5:** The evaluation of autophagosome and autolysosome in liver tissue of ICR mice with GEF-induced liver injury

Parameters	Control	GEF	NG50 + GEF	NG100 + GEF	NG200 + GEF
Autophagosomes	−	+++	++	+	+
Autolysosomes	−	+++	++	+	+

(−) No variation, (+) Small variation, (++) Medium variation, (+++) Big variation

GEF: Gefitinib; NG: Naringin

## Results

***Effect of NG on food intake, body weight, liver weight, and liver index in mice with liver injury***

Compared to the control group, the food intake and body weight of the mice in the GEF groups decreased, but liver weight and liver index increased significantly (*P*<0.05). When compared to the GEF group, mice treated with NG at doses of 200 mg/kg showed an increase in food intake, but a decrease in liver weight(NG100+GEF, NG200+GEF) and liver index(NG50+GEF, NG100+GEF, NG200+GEF), with statistically significant differences (*P*<0.05) (Figure 2 ). The measurements of food intake, body weight, and liver weight are all expressed in grams (g).

***Effects on serum enzymes related to liver function***

Compared to the control group, animals in the GEF group showed a significant increase in ALT, AST, LDH, and ALP activities (*P*<0.05) in serum. In comparison to the GEF group, animals co-treated with GEF and NG showed a significant decrease in ALT, AST, LDH, and ALP activities (*P*<0.05) in serum (Table 1).

***Anti-oxidant effects***

When compared to the control group, animals in the GEF group showed a significant decrease in GSH and SOD activities (*P*<0.05). In comparison to the GEF group, animals co-treated with NG100 and GEF showed a significant increase in GSH, SOD, and CAT activities (*P*<0.05) (Table 2).

***Histopathological findings***

In the control group, liver tissue in mice exhibited well-organized liver cord arrangement and intact hepatocyte morphology. In the GEF group, liver tissue showed disrupted liver cord morphology, extensive focal necrosis of hepatocytes, and infiltration of inflammatory cells. When compared to the GEF group, mice in the NG 50 + GEF group showed slight infiltration of neutrophils in liver tissue, but it still showed sporadic hydropic degeneration of hepatocytes. In the NG100 + GEF group, there was slight infiltration of neutrophils, minor areas of necrotic lesions, sporadic hydropic degeneration of hepatocytes, and relatively orderly liver cord arrangement. In the NG200 + GEF group, there were no significant abnormalities observed in liver tissue, with significantly reduced infiltration of neutrophils and hydropic degeneration, and intact hepatocyte structure (Figure 3). Histopathological findings are evaluated (Table 3).

***Analysis of TUNEL staining results***

To verify whether NG has an inhibitory effect on GEF-induced liver cell apoptosis, TUNEL staining was performed. When cells undergo apoptosis, DNA double-strand or single-strand breaks produce a large amount of sticky 3’-OH ends, which are labeled with fluorescein for apoptosis detection. In this experiment, paraffin sections of mouse liver tissue were used for detection, and the staining sections were observed under an inverted microscope. Compared to the control group, TUNEL results in the GEF group showed an increase in cell apoptosis (green fluorescence). When compared to the GEF group, TUNEL results in the NG50 + GEF, NG100 + GEF, and NG200 + GEF groups showed a gradual decrease in cell apoptosis, with a dose-dependent trend (Figure 4). Therefore, NG has a reversing effect on GEF-induced liver cell apoptosis.

***Immunohistochemical analysis***

To further validate the effects of NG, immunohistochemical staining was used to observe the staining of apoptosis proteins cleaved-caspase3 and PARP1. Immunohistochemistry involves the binding of antigens with antibodies and uses a chromogen to label the antibody for visualization, allowing for qualitative analysis and localization of proteins. In the control group, cleaved-caspase3 and PARP1 in liver tissue were both negative. Compared to the control group, the GEF group showed a significant increase in immunopositivity for cleaved-caspase3 and PARP1. In the treatment groups (NG50, NG100, NG200), there was mild immunopositivity for cleaved-caspase3 and PARP1 when compared to the GEF group (Figure 5, 6). Immunohistochemical findings are evaluated in Table 4.

***Observation of autophagosomes and autolysosomes by transmission electron microscopy***

Autophagy is the process by which cells capture their own cytoplasm and organelles and consume them within lysosomes, a self-devouring process. Autophagosomes serve as the gold standard for demonstrating cellular autophagy. The characteristic morphology of autophagosomes is the formation of a sealed, round, spherical structure. As autophagy progresses, autophagosomes fuse with lysosomes to form autolysosomes, breaking down their inner membrane and its encased material. Transmission electron microscopy results showed that in the control group, liver cell structures were generally well-preserved, with abundant cytoplasm, intact organelle structures, and no noticeable swelling. The cell nuclei were round with intact nuclear membranes, and the perinuclear cleft was normal. Mitochondria were present in sufficient numbers, mostly oval or short rod-shaped, with generally intact structures, membranes, and cristae. When compared to the control group, the GEF group exhibited an increased number of autophagosomes and autolysosomes around the cell nucleus (Figure 7). Transmission electron microscopy findings are evaluated as shown in Table 5 . These results suggest to some extent that GEF induces autophagy in liver tissue cells, and NG has an inhibitory effect on the autophagy induced by gefitinib.

## Discussion

This study aimed to establish an ICR mouse model of GEF-induced liver toxicity to explore the protective effects and mechanisms of NG on GEF-induced liver toxicity. The results showed that NG alleviated GEF-induced liver toxicity by inhibiting cell apoptosis and autophagy.

ALT and AST are important markers for liver cell damage and liver disease diagnosis (16). ALT and AST are mainly distributed in liver tissues, and their levels in normal serum are very low. When liver cells are damaged, ALT and AST are released into the bloodstream, leading to a significant increase in ALT and AST levels in serum (17). The results of this study showed that the activities of liver function-related serum enzymes such as ALT, AST, LDH, and ALP were significantly increased in mice in the GEF group, indicating liver damage. Histopathological slides showed hydropic degeneration of hepatocytes, infiltration of neutrophils, and focal liver necrosis in mice in the GEF group. This is consistent with the findings of previous studies by Shao Jinjin (18). On the other hand, in the NG50 + GEF, NG100 + GEF, and NG200 + GEF groups, treated for 4 weeks, the activities of ALT, AST, LDH, and ALP in the serum were significantly reduced. NG treatment significantly improved the pathological changes in liver tissue. This suggests that NG has a protective effect against GEF-induced liver injury. This finding is consistent with the research results of Akamo *et al*., who found that NG treatment improved liver function in mice treated with sodium diclofenac and cyclophosphamide (10,12).

Under the action of CYP450 3A and 1A enzymes, GEF is metabolized into chemically reactive metabolites, which then bind to GSH (19). Studies have found that mice with depleted GSH when orally administered GEF at 500 mg/kg per day for 4 days, showed nuclear fragmentation and single hepatocyte death in the liver. In these mice, the liver expression levels of heme oxygenase 1 (Hmox1) and metallothionein 2 (Mt2) mRNA, caspase 3/7 enzyme activity, and the amount of 2-thiobarbituric acid reactive substances significantly increased, indicating the presence of oxidative stress. In our study, compared to the control group, mice in the GEF group showed a significant decrease in GSH, and SOD activities in liver tissue (*P*<0.05). Compared to the GEF group, mice co-treated with NG100 and GEF showed a significant increase in GSH, SOD, and CAT activities in liver tissue (*P*<0.05) (20).

There is a close relationship between liver cell apoptosis and drug-induced liver injury (21). Some studies have suggested that GEF-induced liver cell apoptosis plays an important role in the pathogenesis of acute liver injury (22). Increasing evidence suggests that caspase activation occurs not only in programmed cell death during development but also in various human diseases (23). Various stimuli such as Bax, oxidants, Ca^2+^ overload, caspase, and ceramide can trigger the release of cytochrome c and apoptosis-inducing factors from mitochondria. Cytochrome c binds to Apaf-1, triggering the activation of caspase-9, and initiating the caspase cascade reaction, ultimately leading to cell apoptosis (24). Caspase-9 is a key upstream activator of the mitochondrial cascade of caspase (25). Caspase-3 is a key effector molecule in cell apoptosis, controlling the morphological changes of DNA fragments and apoptosis, and is also a key mediator of mitochondrial membrane potential (Δψm) loss. Therefore, inhibiting caspase activity can protect cells from the loss of Δψm (26). Poly ADP-ribose Polymerase (PARP) is a cleavage substrate for effector caspases and its cleavage is considered an important marker of apoptosis (27). We used the TdT-mediated dUTP Nick-End Labeling (TUNEL) method to detect cell apoptosis. The results showed that the green fluorescence was significantly increased in the GEF group. Compared to the control group, and in the NG50 + GEF, NG100 + GEF, and NG200 + GEF groups, TUNEL results showed a gradual decrease in cell apoptosis. These results preliminarily confirmed that NG alleviates GEF-induced liver toxicity by inhibiting liver cell apoptosis. Furthermore, immunohistochemical staining was used to observe the expression of apoptosis-related proteins cleaved-caspase3 and PARP1, confirming whether GEF indeed induces liver cell apoptosis and the anti-apoptotic effect of NG. The immunohistochemical staining for cleaved-caspase3 and PARP1 in the control group was negative. In contrast, the immunohistochemical staining for cleaved-caspase3 and PARP1 in the GEF group and NG50 + GEF, NG 100 + GEF, and NG200 + GEF groups was positive. The quantity of cleaved-caspase3 and PARP1 immunohistochemical staining in the NG50 + GEF, NG100 + GEF, and NG200 + GEF groups decreased with increasing drug doses. NG inhibits apoptosis by reducing cleaved-caspase3 expression.

Researchers have observed that cell autophagy occurs concurrently with cell apoptosis in GEF-induced liver cell apoptosis(19). Autophagosomes and autolysosomes are indicative of increased autophagic activity (28). Electron microscopy results showed typical membrane structures in the livers of mice in the GEF group, including black granular or indistinctly clustered autophagosomes and autolysosomes, suggesting that GEF may induce autophagy in liver cells. In the NG50 + GEF, NG100 + GEF, and NG200 + GEF groups, there was a decreasing trend in the number of autophagosomes and autolysosomes with increasing NG concentration. Our research indicates that NG has an inhibitory effect on GEF-induced autophagy at certain concentrations. 

Studies have found that NG inhibits apoptosis and autophagy by reducing the expression of caspase-3 and the level of Light chain 3B (LC3B), thus alleviating CYCP-induced hepatotoxicity, which is consistent with this result (11).

## Conclusion

In summary, the *in vivo* experiments confirm that NG has a protective effect against liver damage induced by GEF in mice. The mechanism appears to involve NG’s ability to have anti-oxidation and inhibit both apoptosis and autophagy, thus alleviating the hepatotoxicity induced by gefitinib. However, further clinical research is needed to evaluate the effectiveness and safety of NG in humans. Specific molecular mechanisms and signaling pathways require further investigation.
